# Injectable Alginate-Peptide Composite Hydrogel as a Scaffold for Bone Tissue Regeneration

**DOI:** 10.3390/nano9040497

**Published:** 2019-04-01

**Authors:** Moumita Ghosh, Michal Halperin-Sternfeld, Itzhak Grinberg, Lihi Adler-Abramovich

**Affiliations:** Department of Oral Biology, The Goldschleger School of Dental Medicine, Sackler Faculty of Medicine, Tel Aviv University, Tel Aviv 6997801, Israel; moumita.ghosh1986@gmail.com (M.G.); michal4@mail.tau.ac.il (M.H.-S.); tzakhi@gmail.com (I.G.)

**Keywords:** regenerative medicine, scaffolds, nanomaterials, extracellular matrix, hydrogels, self-assembly

## Abstract

The high demand for tissue engineering scaffolds capable of inducing bone regeneration using minimally invasive techniques prompts the need for the development of new biomaterials. Herein, we investigate the ability of Alginate incorporated with the fluorenylmethoxycarbonyl-diphenylalanine (FmocFF) peptide composite hydrogel to serve as a potential biomaterial for bone regeneration. We demonstrate that the incorporation of the self-assembling peptide, FmocFF, in sodium alginate leads to the production of a rigid, yet injectable, hydrogel without the addition of cross-linking agents. Scanning electron microscopy reveals a nanofibrous structure which mimics the natural bone extracellular matrix. The formed composite hydrogel exhibits thixotropic behavior and a high storage modulus of approximately 10 kPA, as observed in rheological measurements. The in vitro biocompatibility tests carried out with MC3T3-E1 preosteoblast cells demonstrate good cell viability and adhesion to the hydrogel fibers. This composite scaffold can induce osteogenic differentiation and facilitate calcium mineralization, as shown by Alizarin red staining, alkaline phosphatase activity and RT-PCR analysis. The high biocompatibility, excellent mechanical properties and similarity to the native extracellular matrix suggest the utilization of this hydrogel as a temporary three-dimensional cellular microenvironment promoting bone regeneration.

## 1. Introduction

Bone defects or fractures can result from trauma, neoplasm, congenital defects, or bone-associated diseases [1]. To date, various bone substitutes, including autografts, allografts and xenografts, are used to treat bone defects, however, they have several drawbacks, including limited availability and potential immunogenicity and disease transmission, respectively [2,3]. Moreover, augmentation using these bone substitutes requires an invasive surgical procedure which may often lead to further bone loss [4,5].

Bone tissue engineering and regenerative medicine aim to restore the architecture and function of the damaged bone tissue using biodegradable scaffolds which serve as temporary matrices to support and direct bone regeneration [6]. Efforts are made towards the development of new injectable biomaterials due to their ability to ameliorate bone defects with irregularly shaped geometry using a minimally invasive route [7]. Hydrogels, either synthetic or natural in composition, are attractive materials for tissue engineering [8,9,10,11]. Particularly, naturally derived polysaccharides, such as chitosan [12], hyaluronic acid [13,14,15] and alginate [16,17,18,19], have been widely explored for tissue engineering purposes due to their inherent biocompatibility, high water content, and characteristics similar to the bone natural extracellular matrix (ECM) [20,21,22]. These unique properties render them easily biodegradable and favorable for cell incorporation and migration. However, their poor mechanical strength compared to that of the natural bone ECM, rapid and unpredictable biodegradation (either enzymatic for chitosan [23] and hyaluronic acid [24] or acid catalyzed hydrolysis and alkali catalyzed β-elimination for alginates [25]), and possible loss of biological properties during fabrication and storage limit their potential for bone regeneration [22,26]. Consequently, natural polysaccharides are often chemically or physically modified to form new controllable and reproducible materials toward biomedical applications [22,25,27,28].

The traditional polysaccharide-based hydrogels are chemically cross-linked by covalent bonds or physically cross-linked by non-covalent interactions [29]. Another way to fabricate polysaccharide-based hydrogels with improved mechanical properties is to combine them with self-assembling peptides [13,30,31,32]. Self-assembling peptides can give rise to mechanically stable hydrogels [33,34,35,36,37,38,39,40], which are typically formed via non-covalent interactions, such as hydrogen-bonding, van der Waals interactions, π–π stacking and electrostatic interactions [41,42,43]. Self-assembling peptides have recently gained increasing interest as promising scaffold materials for tissue engineering applications [44,45,46,47,48,49]. They self-organize from basic building blocks into supramolecular structures, mimicking the native ECM, under mild conditions. Moreover, they are easily synthesized and their properties can be tuned through changes at the sequence level. Finally, they are biocompatible, biodegradable and can be injectable, thus easily adopting the architecture of the tissue defect [50]. A large number of studies have focused on fluorenylmethoxycarbonyl (Fmoc)-modified oligopeptides and their ability to form hydrogels [42,51], mainly due to the intrinsic propensity of the Fmoc moiety to rapidly self-assemble through π–π and hydrophobic interactions. A notable example of Fmoc-based hydrogels is the Fmoc-diphenylalanine (FmocFF) peptide that efficiently assembles to form a rigid nanofibrous hydrogel under physiological conditions [52,53]. FmocFF has been combined with different polysaccharides to form new composite hydrogels for different biomedical applications, including cell culture [31,54,55] and drug delivery [13,54,56,57].

Alginate/FmocFF composite hydrogels were recently explored by several groups for tissue engineering and drug delivery applications [54,55,57]. Alginates are natural anionic biopolymers derived from brown seaweeds and are composed from 1,4-linked β-d mannuronic acid (M) and α-l-glucoronic acid (G) units. The ability of alginates to from hydrogels in the presence of calcium ions is the basis for a wide variety of applications, such as wound dressing materials [58], three-dimensional culture [59], cell and protein delivery [60,61,62], and cardiac regeneration [17,63,64]. In 2016, Xie et al. developed a new FmocFF/alginate composite hydrogel, formed by a calcium ion trigger, for drug delivery [57]. Calcium ions triggered both the self-assembly of FmocFF and the cross-linking of alginate to form hybrid beads which facilitated the controlled release of docetaxel, as determined by the change of the concentrations of alginate and FmocFF. Another FmocFF/alginate hydrogel was reported in 2016 by Celik et al. for chondrocytes culture [54]. In this study, FmocFF was self-assembled in the alginate solution, followed by ion cross-linking of alginate using varying concentrations of calcium chloride. The cross-linked hydrogels showed higher mechanical properties and stability to degradation as the calcium chloride concentration increased, up to 0.25%. The hydrogels were biocompatible and supported the viability of chondrocytes for 14 days. Gong et al. also formed a composite FmocFF/alginate hydrogel, but without the addition of cross-linking molecules [55]. This hydrogel showed different morphologies resulting from the change in sodium alginate concentration. The composite hydrogels were biocompatible and supported the growth of epithelial cells. Moreover, they showed higher mechanical properties and resistance to proteases degradation compared to FmocFF alone.

Alginates have been widely investigated as potential scaffolds for bone regeneration [65]. To this end, alginates are physically cross-linked with calcium or other divalent cations. It has been reported, however, that in physiological solution, cross-linked alginate hydrogels show weak mechanical properties within a few hours [65]. To overcome this issue, alginates are chemically modified by acetylation, phosphorylation, sulfation, and esterification [66,67,68,69]. The combination of cross-linked alginate with bone ceramics has been also proposed as a route to improve the mechanical properties of alginate, resulting in osteogenic composite organic-inorganic injectable or spongy scaffolds, in which the bone ceramics support and reinforce the alginate [70,71,72,73].

Here, we investigate the recently described technique of incorporating a self-assembling peptide within alginate to form an injectable hydrogel for bone regeneration. We exploit the ability of self-assembling peptides in a composite material to mimic the nanofibrillary nature of the ECM, and to tailor its mechanical properties to the desired stiffness and strength. We demonstrate that the biocompatible hydrogel allows MC3T3-E1 preosteoblasts to proliferate and differentiate into bone forming cells, resulting in calcifications on the hydrogel surface. 

## 2. Materials and Methods 

### 2.1. Materials

Lyophilized Fmoc-Phe-Phe-OH (FmocFF) was purchased from Bachem (Budendorf, Switzerland). Sodium alginate, 3-[4,5-dimethylthiazole-2-yl]-2,5-diphenyltetrazolium bromide (MTT), fluorescein diacetate, propidium iodide, 4-Methylumbelliferyl phosphate (4-MUP), Rhodamin B, and Alizarin red were purchased from Sigma Aldrich (Rehovot, Israel). MC3T3-E1 preosteoblast cells were purchased from ATCC (Manassas, VA, USA). Pure Link RNA Mini Kit was purchased from Invitrogen; Thermo Fisher Scientific (Carlsbad, CA, USA), qScript cDNA Synthesis Kit and SYBR green reagents with Perfecta SYBR Green FastMix reagent with specific gene primers were purchased from Quanta Biosciences (Boston, BA, USA).

### 2.2. Methods

#### 2.2.1. Preparation of Alginate/FmocFF Composite Hydrogel

FmocFF stock solution was prepared in dimethyl sulfoxide (DMSO) solvent. 100 mg of the powdered peptide was dissolved in 1 mL DMSO using vortex until a transparent solution was obtained. 25 µL of the peptide stock solution was added to 975 µL double distilled (dd) water followed by immediate vortexing to obtain pure FmocFF hydrogel. For the preparation of Alginate/FmocFF composite hydrogel, sodium alginate powder was gradually dispersed in dd H_2_O (4 mg/mL) while stirring, and then stirred for 12 h at room temperature until full dissolution. Next, 25 µL of FmocFF solution in DMSO was added to 975 µL of the alginate solution (4 mg/mL), immediately followed by vortex mixing. The final concentration of the FmocFF in the hydrogel was 2.5 mg/mL.

#### 2.2.2. Scanning Electron Microscopy (SEM)

Samples of FmocFF, sodium alginate and Alginate/FmocFF composite hydrogel were placed on glass coverslips and left to air dry at ambient conditions. The samples were then coated with Au for conductance and viewed using a scanning electron microscope (JEOL, Tokyo, Japan) operating at 20 kV.

#### 2.2.3. Rheological Measurements

Rheological analysis was performed using an AR-G2 controlled-stress rheometer (TA Instruments, New Castle, DA, USA). In order to determine the linear viscoelastic region, oscillatory strain (0.01–100%), frequency sweep (0.01–100 Hz), and sheer rate (0.01–100/s) tests were performed in parallel plate geometry on 320 µL of freshly prepared hydrogel solution and the alginate solution (resulting in a gap size of 0.6 mm), at room temperature. Time sweep oscillatory tests were performed for 24 h at a constant frequency of 5 Hz and strain of 0.5% to determine G’ and G”, the storage and loss moduli, respectively, for each sample. Thixotropic study was performed to examine the recovery behavior of the hydrogel. The recovery of the G’ value of the destroyed gel was monitored at a constant frequency of 5 Hz.

#### 2.2.4. Cell Viability on the Alginate/FmocFF Composite Hydrogel

Murine MC3T3-E1 preosteoblast cells were cultured in Alpha-Minimum Essential Medium (α-MEM) supplemented with 10% fetal calf serum, 100 U·mL^−1^ penicillin, and 100 U·mL^−1^ streptomycin in a petri dish at 37 °C in a humidified atmosphere in an incubator containing 5% CO_2_. Hydrogels were formed in a 96-well plate and washed with culture medium several times over 2 days to ensure complete removal of excess materials and DMSO, followed by UV sterilization for 30 min. Then, cells were seeded on the prewashed hydrogels and left at 37 °C in a humidified atmosphere containing 5% CO_2_. The differentiating cells were supplemented with differentiation medium containing ascorbic acid and beta-glycerophosphate every two days for 14 days. Finally, cell viability was assessed using the MTT assay 3 days after seeding for the non-differentiating cells, as well as at 14 days, following osteogenic differentiation. MTT stock solution (5 mg/mL) was prepared in phosphate buffer saline (PBS). Addition of 20 μL from this solution was done to each well followed by a 4-h incubation. 100 μL DMSO was added to extract the MTT reduced adduct (Formazan) formed in each well. The plates were placed on a shaker for 20 min to allow a complete dissolution of the precipitated formazan in DMSO. Finally, absorbance was measured from each well using Tecan Spark plate reader at 570 nm wavelength. Background was corrected at 680 nm. The results were presented as the percentage of viable cells with respect to control cells on the same plate. 

The qualitative assessment of cell viability on the composite hydrogel was performed using the Live/Dead staining before and after osteogenic differentiation. First, hydrogels were prepared in a 24-well plate and repeatedly rinsed with culture medium, followed by UV sterilization, for 2 days. Next, cells were seeded on the hydrogels for 3 and 14 days for viability assessment before and after differentiation, respectively. The differentiating cells were supplemented with osteogenic medium containing differentiation factors every 2 days, for a total duration of 3 or 14 days. Following 3 or 14 days of cell growth, as described above, the medium was removed from each well. A Live/Dead staining solution containing fluorescein diacetate (6.6 µg/mL) and propidium iodide (5 µg/mL) was then used to visualize the proportion of viable versus non-viable cells on the composite hydrogel, respectively. The labelled cells were immediately viewed using a Nikon Eclipse Ti fluorescent microscope and images were captured by a Zyla scMOS camera using Nikon Intensilight C-HGFI fluorescent lamp. 

To further assess the adherence of cells to the Alginate/FmocFF composite hydrogel, hydrogels were prepared in a glass-bottom 96-well plate and repeatedly rinsed with culture medium, followed by 30 min UV sterilization, for 2 days. Next, cells were seeded on the hydrogels for 3 days and incubated with a dye mixture containing fluorescein diacetate (6.6 µg/mL) and 10^−6^ wt.% rhodamin B to identify the cells and the composite hydrogels, respectively, for 5 min at room temperature, followed by imaging with Leica SP8 X Confocal Microscope.

#### 2.2.5. Alkaline Phosphatase (ALP) Activity

To determine the intracellular ALP activity, hydrogels were formed in a 96-well plate and repeatedly rinsed with culture medium, followed by 30 min UV sterilization, for 2 days. Next, 10,000 MC3T3-E1 preosteoblasts were seeded on the prewashed hydrogels and supplemented with differentiation medium every 2 days for a period of 14 days. After 14 days, the hydrogels were stained with 100 µL ALP substrate solution containing 4-Methylumbelliferyl phosphate (4-MUP) and incubated for 30 min in the dark. Finally, fluorescence was measured with excitation at 360 nm and emission at 440 nm.

#### 2.2.6. Mineralization Assay 

The extent of matrix mineralization of MC3T3-E1 preosteoblasts on the Alginate/FmocFF hydrogel was evaluated by Alizarin red staining assay which quantifies the amount of mineralization arising from bone nodule formation. The hydrogels were formed in a 24-well plate and repeatedly washed with culture medium, followed by UV sterilization for 30 min, for 2 days. 50,000 MC3T3-E1 preosteoblasts were seeded on the prewashed hydrogels and supplemented with differentiation medium every 2 days for a period of 14 days. The amount of induced calcification was quantified by Alizarin red staining. After washing off excessive dyes, optical light images were acquired from each well. The percentage of calcification was quantified by Image J analysis from each well before and after differentiation and normalized to the cell count.

#### 2.2.7. Reverse Transcription-Quantitative Polymerase Chain Reaction (RT-qPCR) 

The relative expression of osteogenic marker genes for MC3T3-E1 preosteoblasts seeded on the Alginate/FmocFF hydrogel, before and after differentiation, was evaluated by RT-qPCR analysis. The differentiating cells cultured on the hydrogels in T-25 culture flasks were supplemented with differentiation medium containing ascorbic acid and beta-glycerophosphate every 2 days for 14 days. Total RNA was extracted using Pure Link RNA Mini Kit according to the manufacturer’s protocol, before and after differentiation. Subsequently, purified total RNA was reverse-transcribed using qScript cDNA Synthesis Kit with random primers according to manufacturer’s protocol. Real-time PCR was performed using SYBR green reagents with Perfecta SYBR Green FastMix reagent with specific gene primers according to manufacturer’s protocol. The expression of target genes, including Runt Related Transcription Factor 2 (Run×2), ALP, osteocalcin (OCN), bone morphogenic protein 2 (BMP-2) and collagen type 1 (COL-1), was normalized relative to the Actin housekeeping gene used as reference by the ΔΔ*C*t method. Data was analyzed with Quant Studio 12K Flex Real Time PCR System v1.2.2 Software (Applied Biosystem: Thermo Fisher Scientific (Carlsbad, CA, USA)). Results were obtained from two series of experiments performed in quadruple repeats of each gene. Primers used for amplification are listed in Supplementary Table S1.

#### 2.2.8. Statistical Analysis

All experiments were repeated two times in quadruple repeats of each gene and the results are expressed as the means ± standard error of the mean. Statistical analysis was performed using Microsoft Excel. The statistical analysis of differences between groups post-differentiation was performed with a significance *p* < 0.05 compared to pre-differentiation in quadruple repeats of each gene in duplicate experiments determined by one-way ANOVA. *p* < 0.05 was considered a statistically significant difference.

## 3. Results and Discussion

### 3.1. Preparation and Characterization of Alginate/FmocFF Composite Hydrogel 

**Figure 1:**
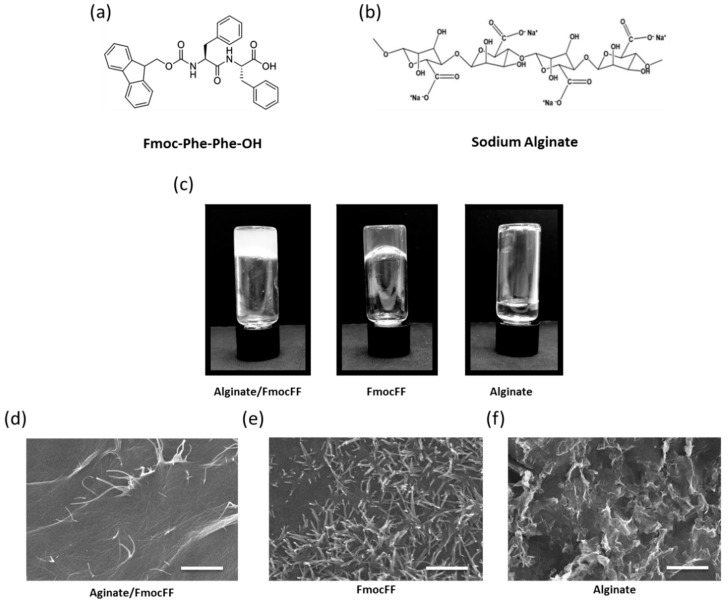
Fabrication and characterization of the Alginate/fluorenylmethoxycarbonyl-diphenylalanine (FmocFF) composite hydrogel. (**a**) Molecular structure of the FmocFF peptide. (**b**) Molecular structure of typical sodium alginate repeated units. (**c**) Inverted vials of the Alginate/FmocFF composite hydrogel and its pure components. (**d**–**f**) SEM micrographs of (**d**) Alginate/FmocFF composite hydrogel, (**e**) Pure FmocFF hydrogel, and (**f**) Sodium alginate solution. Scale bar = 5 µm.

To form Alginate/FmocFF composite hydrogel, FmocFF (Figure 1a) was incorporated in the sodium alginate polymeric material (Figure 1b) by the solvent switch method to a final concentration of 0.4 wt.% sodium alginate and 0.25 wt.% FmocFF [55]. Within a few minutes, a stable hydrogel was formed (Figure 1c). Pure FmocFF hydrogel at a concentration of 0.25 wt.% was also prepared by the solvent switch method in DMSO and water solution. While 0.25 wt.% FmocFF alone formed a stable transparent hydrogel at room temperature, 0.4 wt.% sodium alginate did not form a hydrogel (Figure 1c). As presented in Figure 1c, the composite hydrogel was more opaque than the pure FmocFF hydrogel. 

SEM analysis was carried out to characterize the morphology of the composite hydrogel and its pure components (Figure 1d–f and Figure S1a,b). SEM images demonstrated that the Alginate/FmocFF composite hydrogel formed long entangled fibrils several micrometers in length, similar to those seen in the pure FmocFF hydrogel at the same concentration (Figure 1d,e). The nanofibrous architecture of the composite hydrogel resembled the fibrillary nature of the ECM, which is a prerequisite when designing scaffolds for tissue engineering [74], as cells are constantly sensing the ECM, interacting, remodeling and migrating through to perform normal functions. Herein, the mixture of alginate and FmocFF ideally mimics glycosaminoglycans and fibrous proteins (e.g., collagen, elastin, fibronectin, and laminin), respectively, the two main macromolecules comprising the ECM [75]. Sodium alginate probably serves as a matrix to immobilize FmocFF molecules, thereby inducing the formation of a composite hydrogel. The presence of a large number of hydroxyl groups probably led to the supramolecular arrangement of FmocFF within the alginate matrix due to the sufficient hydrogen bonding among the building blocks and water [55].

### 3.2. Rheological Characterization

**Figure 2:**
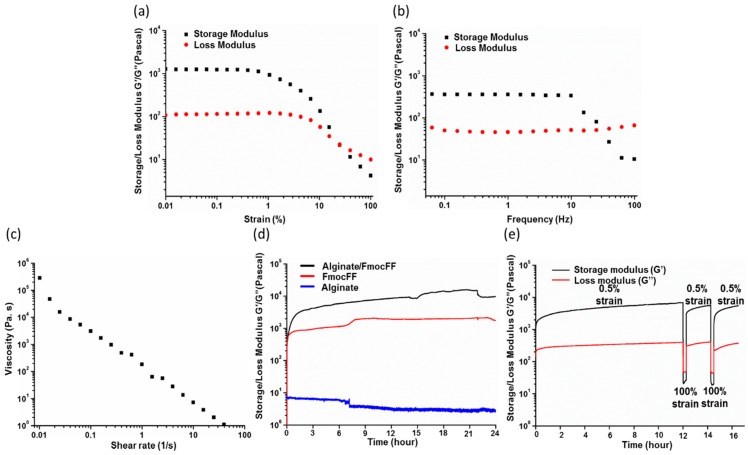
Rheological characterization of the hydrogels. (**a**) Strain sweep of the Alginate/FmocFF composite hydrogel. (**b**) Frequency sweep of the Alginate/FmocFF composite hydrogel. (**c**) Viscosity versus shear rate of the Alginate/FmocFF composite hydrogel. (**d**) In situ time sweep oscillation measurements of hydrogels formation by pure alginate, FmocFF, and Alginate/FmocFF composite gel for 24 h. (**e**) G’ and G” of Alginate/FmocFF composite hydrogel on time sweep (0–120 min) and subsequent step strain (0.5% and 100%) measurements.

In order to study the kinetics of the hydrogel formation and their mechanical properties, rheological analysis was performed. First, we measured the G’ and G” of the composite hydrogel at dynamic strain sweep (at 5 Hz frequency). To observe the effect of oscillatory strain, the hydrogels were subjected to 0.01–100% strain sweep at a constant frequency, resulting in a wide linear viscoelastic region of up to 10% strain (Figure 2a). Frequency sweep experiments at constant strain using a frequency range of 0.1–100 Hz also showed a wide linear viscoelastic region (Figure 2b). Both G’ and G” were frequency-independent throughout the frequency range of 0.1–100 Hz, implying the formation of an entangled fibrillary network throughout this frequency range [76]. Figure 2c shows the variation of viscosity of the composite gel with respect to shear rate (0.01–100/s). The composite hydrogel showed shear-induced breakage, supporting a shear-thinning behavior of the gel. The high viscosity value of the composite hydrogel at low shear rate might have stemmed from the entangled networks [77]. With the enhancement in shear rate, these entanglements were disrupted by the imposed deformation, resulting in the breakage of the fibril network and depletion of the gel. Based on both the dynamic strain sweep and frequency sweep tests, we determined the linear viscoelastic region and performed a time sweep measurement at a fixed strain of 0.5% and frequency of 5 Hz, over 24 h. The Alginate/FmocFF composite hydrogel reached a G’ value of 9993 Pa, while the G’ of the pure FmocFF was 1762 Pa, indicating the effect of the combination of FmocFF with sodium alginate on the mechanical properties of the composite material, resulting in a 5.67-fold increase in the G’ (Figure 2d). Sodium alginate alone had a very low G’ value (3 Pa), indicating no gel formation (Figure 2d). As a strong gelator, FmocFF is hypothesized to be the driving force of the supramolecular organization with sodium alginate, thus producing a composite hydrogel with higher rigidity compared to the individual components. We have recently demonstrated a similar effect on the rigidity of the FmocFF hydrogel using Hyaluronic acid. In that case, the combination of Hyaluronic acid and FmocFF in a 1:3 ratio resulted in a G’ value 2.8 times higher than that of the pure FmocFF [13]. 

Interestingly, the gelation of the pure FmocFF hydrogel was attained within less than 5 min, while the gelation of the Alginate/FmocFF composite required approximately 60 min (Figure 2d). The G’ value of the Alginate/FmocFF composite hydrogel was found to be sufficiently higher than G”, indicating the formation of a viscoelastic composite hydrogel (tanδ < 1) (Figure S2). The kinetics of hydrogel formation shows that the FmocFF peptide rapidly self-organizes to form a hydrogel. In contrast, the composite hydrogel requires longer time periods to reach the viscoelastic equilibrium. These results indicate that the composite system is characterized by enhanced mechanical properties compared to the pure FmocFF and sodium alginate alone under the same conditions. 

Hydrogels exhibiting a thixotropic property, i.e., reversible sol–gel transformations upon exposure to mechanical stress, are an intriguing class of supramolecular systems with potential applications as injectable biomaterials. Interestingly, the Alginate/FmocFF hydrogel showed a recovery of mechanical strength after the large amplitude oscillatory breakdown with 100% strain (Figure 2e). The initial G’ value of about 6982 Pa dropped down to 720 Pa under a large amplitude oscillatory force 100% strain and 5.0 Hz frequency, indicating the transformation of the gel to a sol state (Figure 2e). As the amplitude oscillation was decreased to 0.5% at the same frequency of 5.0 Hz, it regained its gelation property, and the G’ value increased to about 5661 Pa, representing the recovery of a gel state, which under repeated 100% amplitude oscillatory force strain and 5.0 Hz frequency, again dropped down to a value of 855 Pa, followed by recovery to 5566 Pa, indicating the thixotropic property of the gel (Figure 2e). Supramolecular hydrogels that self-heal spontaneously after damage are of particular interest [78]. This trait extends the lifetime of a material and makes it an ideal candidate for tissue engineering applications involving mechanical stress or injection [9,79]. The hydrogel injectability characteristic, i.e., its self-healing behavior, results from the dynamic nature of reversible molecular interactions, which results in shear thinning behavior (decrease in viscosity as shear stress increases). This characteristic is crucial and of utmost importance when designing biomaterials for minimally invasive surgery techniques. 

### 3.3. Biocompatability of the Alginate/FmocFF Composite Hydrogel

To further study the potential of the composite hydrogel to be used as a scaffold for bone tissue engineering applications, we tested its biocompatibility using in vitro cell culture experiments. MC3T3-E1 preosteoblast cells were seeded on the Alginate/FmocFF composite hydrogel. The viability of the MC3T3-E1 cells was evaluated by MTT assay after 3 days and 14 days, following osteogenic differentiation. Figure S3a shows high biocompatibility of 92% and 89% viable cells on the Alginate/FmocFF composite hydrogel at the two timepoints, respectively. 

**Figure 3:**
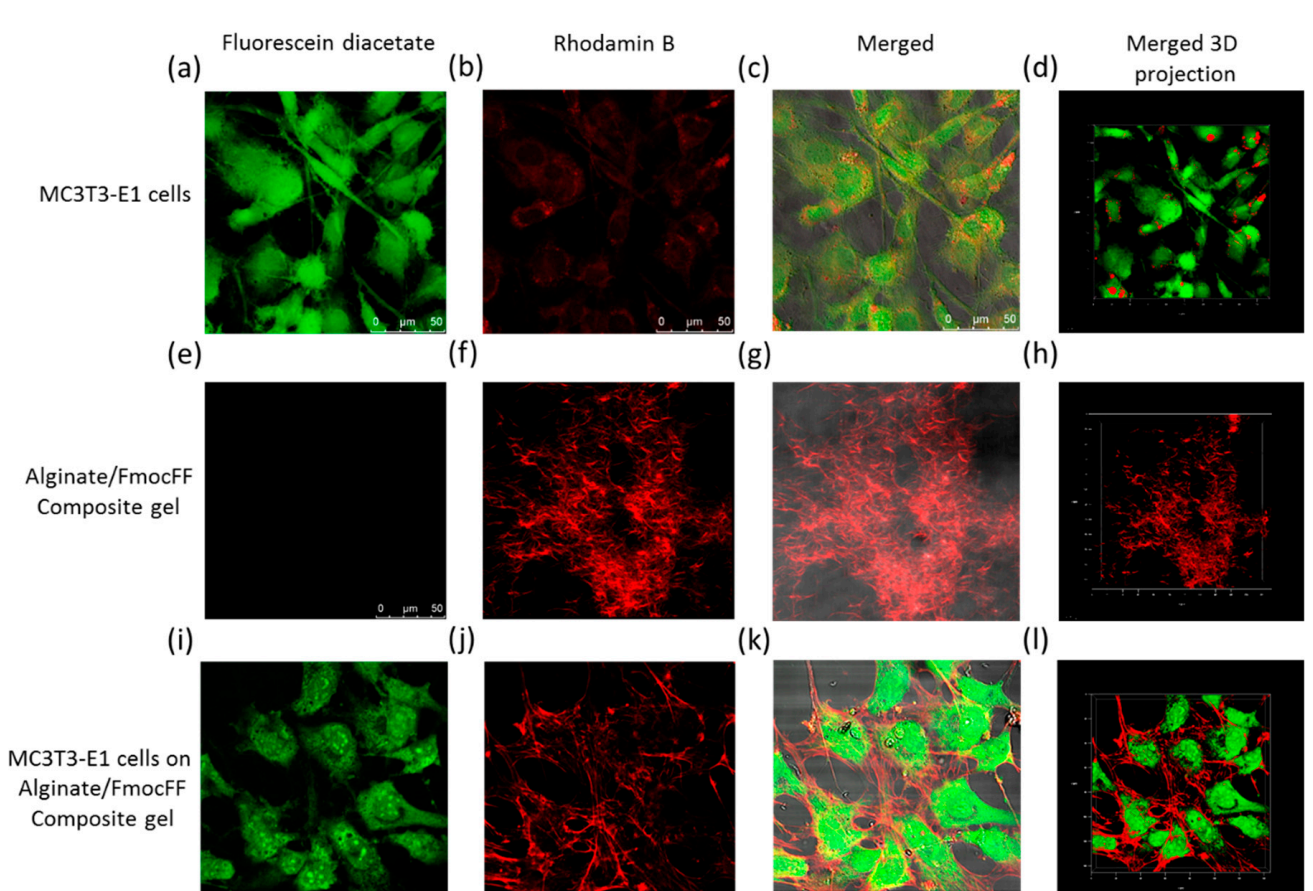
MC3T3-E1 cell viability and spreading onto Alginate/FmocFF composite hydrogel. (**a**–**d**) Control MC3T3-E1 cells stained with fluorescein diacetate and rhodamin B after 3 days of culture on a plate. (**e**–**h**) control Alginate/FmocFF hydrogel stained with fluorescein diacetate and rhodamin B. (**i**–**l**) MC3T3-E1 cells cultured for 3 days on Alginate/FmocFF composite hydrogel and stained with fluorescein diacetate and rhodamin B. Scale bar = 50 µm.

In addition, the cells were stained after 3 days and 14 days using Live/Dead staining comprising fluorescein diacetate, a cell membrane dye used to indicate live cells (green), and propidium iodide, a DNA stain which indicates dead cells (red). Figure S3b shows the Alginate/FmocFF composite hydrogel to be highly populated with green cells. In addition, no propidium iodide staining could be detected on the Alginate/FmocFF composite hydrogel, implying that the hydrogel supports the viability of MC3T3-E1 cells. Moreover, confocal microscopy was used to image the 3D structure of the hydrogel embedded with the cells. Figure 3 shows MC3T3-E1 cells spread on the Alginate/FmocFF composite hydrogel. Furthermore, the integration of fluorescein diacetate (green) stained MC3T3-E1 cells in the rhodamin B (red) stained gel matrix could be observed, demonstrating the elongation of the cell directed by the hydrogel fibrillary structure (Figure 3i–l). As controls, cells cultured directly on the plate showed high intensity of fluorescein diacetate (green) and low rhodamin B (red) staining (Figure 3a–d), and stained Alginate/FmocFF composite hydrogel showed high intensity of rhodamin B (red) staining, without any fluorescein diacetate (green) (Figure 3e–h).

### 3.4. Osteogenesis on the Alginate/FmocFF Composite Hydrogel 

**Figure 4:**
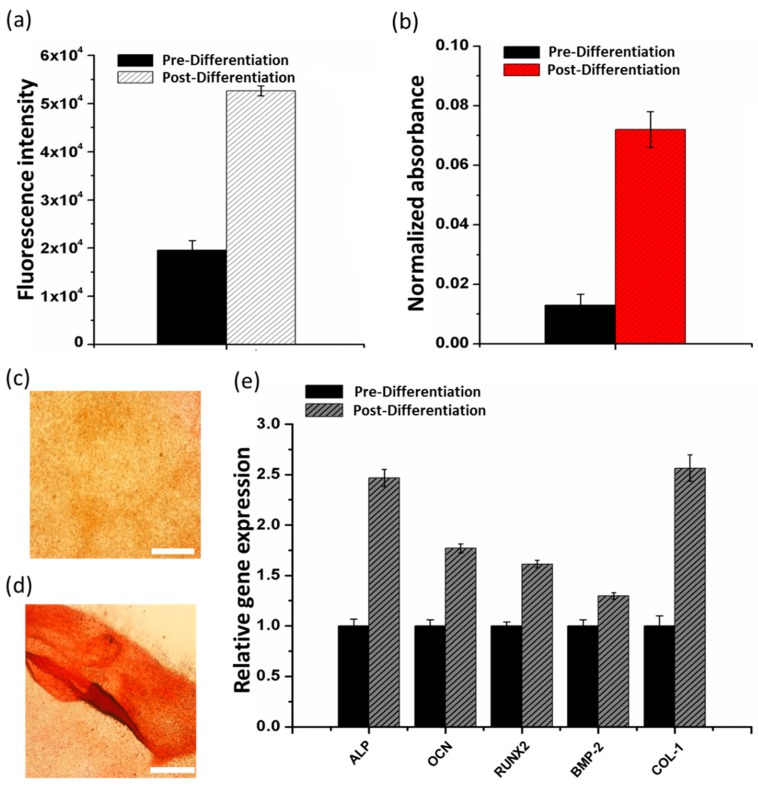
Osteogenesis on the Alginate/FmocFF composite hydrogel. (**a**) Quantification of Alkaline Phosphatase (ALP) activity of MC3T3-E1 preosteoblast cells 3 days after seeding on Alginate/FmocFF composite hydrogel and following 14 days of osteogenic differentiation. (**b**) Quantification of calcification by Alizarin red staining of MC3T3-E1 preosteoblast cells 3 days after seeding on Alginate/FmocFF composite hydrogel and following 14 days of osteogenic differentiation. (**c**,**d**) Optic microscope images of MC3T3-E1 preosteoblast cells stained with Alizarin red (**c**) 3 days after seeding on Alginate/FmocFF composite hydrogel and (**d**) following 14 days of osteogenic differentiation. Scale bar = 500 µm. (**e**) Relative mRNA expression of osteogenic genes (ALP, OCN, RUNX2, BMP-2, and Col-I) was quantitated by RT-qPCR analysis of MC3T3-E1 cells 3 days after seeding on Alginate/FmocFF composite hydrogel and following 14 days of osteogenic differentiation.

To study the potential of the Alginate/FmocFF composite hydrogel to serve as a scaffold that can induce osteoblast differentiation, we measured the ALP activity of the cells that were grown on the composite hydrogel. ALP is an important early osteogenic differentiation marker of progenitor cells. To determine the activity of ALP, MC3T3-E1 preosteoblasts were seeded on the hydrogels for 14 days. After 14 days, ALP activity was measured by adding an ALP substrate, 4-Methylumbelliferyl Phosphate (4-MUP), to the cells (Figure 4a). The increase in ALP activity observed after 14 days of differentiation compared to pre-differentiated cells suggests that the Alginate/FmocFF composite hydrogel is capable of inducing osteogenic response to preosteoblast cells.

In addition, during mineralization, osteoblasts produce extracellular calcium deposits, which can be detected by Alizarin red staining that quantifies the amount of mineralization arising from bone nodule formation. The extent of matrix mineralization of MC3T3-E1 preosteoblasts grown on the Alginate/FmocFF hydrogel was quantified by Alizarin red staining assay before and after differentiation. The staining was normalized to the number of cells. As shown in Figure 4b, the quantified intensity of Alizarin red staining was higher in differentiated cells, as compared to the pre-differentiated cells. In addition, Figure 4c,d shows that the cells were stained more intensely after 14 days incubation on Alginate/FmocFF hydrogel. These results provide convincing evidence that the Alginate/FmocFF hydrogel efficiently induces the differentiation and mineralization of MC3T3-E1 preosteoblast cells.

Differentiation of osteoblasts has been previously shown to affect bone formation [80]. The differentiation process includes three main stages, namely cell proliferation, ECM production and mineralization of the ECM [80,81]. These three stages are associated with the expression of typical markers of osteogenic differentiation, including Run × 2, ALP, OCN, BMP-2 and COL-1 [14,81,82]. To further investigate the ability of the Alginate/FmocFF composite hydrogel to support osteogenic differentiation, we determined the relative mRNA expression levels of these genes. Total RNA was extracted from cells cultured on the composite hydrogel before and after 14 days of differentiation, followed by RT-qPCR analysis. Actin was used as a house-keeping normalizing gene. Significance level of *p* < 0.05 compared to pre-differentiation was determined by one-way ANOVA. The significantly elevated expression of ALP and COL-1 and moderate to high expression of OCN and RUNX2 (Figure 4e) suggest that the Alginate/FmocFF hydrogel supports successful proliferation and early maturation of preosteoblasts to osteoblasts. 

Proliferation leads to subsequent induction of genes associated with matrix maturation and mineralization, which is supported by a temporal sequence of events in which enhanced expression of ALP and increased expression of OCN is observed as the preosteoblasts differentiate into mature osteoblasts. Further, enhanced levels of COL-1 indicate the mineralization of the matrix. It has been previously reported that BMP-2 plays a significant role in stimulating the differentiation of osteoblasts by stimulating the Smad signaling pathway, which regulates the transcription of osteogenesis-related genes, such as ALP, COL-1, OCN and RUNX2 [83,84]. Indeed, elevated expression levels of BMP-2 could be observed (Figure 4e). Thus, the increase in osteoblastic markers expression confirmed the differentiation of preosteoblasts grown on the Alginate/FmocFF hydrogel scaffold. Moreover, this observation is in line with both the calcium staining of the mineralized matrix after differentiation and the elevation in ALP activity. The fibrillar morphology of the composite hydrogel, along with its increased stiffness, probably allowed cellular adhesion and diffusion of osteogenic factors to the adhered preosteoblast cells, thus promoting further differentiation into osteoblasts [85].

## 4. Conclusions

In this study, we propose the application of a composite Alginate/FmocFF hydrogel as an injectable scaffold for bone regeneration. The hydrogel was fabricated by self-assembly of FmocFF in a sodium alginate aqueous solution. This platform exploits the similarity of sodium alginate and FmocFF to glycosaminoglycans and fibrous proteins, respectively, which are the main macromolecules composing the ECM. The resulting hydrogel exhibits thixotropic behavior and a high storage modulus of approximately 10 kPa. The high mechanical properties may be attributed to the supramolecular organization derived by FmocFF within the sodium alginate solution. Moreover, the composite hydrogel facilitates the adhesion, proliferation and osteogenic differentiation of MCT3T-E1 preosteoblasts. The high biocompatibility, excellent mechanical properties and similarity to the native ECM strongly suggest that the Aginate/FmocFF composite hydrogel platform holds promise for effective bone regeneration. Further studies should assess the in vivo effect of the hydrogel on bone formation in critical size bone defects, as well its degradation process. 

## Supplementary Materials

The following are available online at https://www.mdpi.com/2079-4991/9/4/497/s1, Figure S1: Scanning electron microscopy micrographs of Alginate/FmocFF composite hydrogel, Figure S2: In situ time sweep oscillation measurements of Alginate/FmocFF composite hydrogel, Figure S3: Viability and Live-Dead staining of MC3T3-E1 cells on the Alginate/FmocFF composite hydrogel, Table S1: Primers used for PCR amplification.
