# Supplementary material for: Injectable Alginate-Peptide Composite Hydrogel as a Scaffold for Bone Tissue Regeneration
Source: Nanomaterials (Basel). 2019 Apr 1;9(4):497. doi: 10.3390/nano9040497 (PMC6523611; doi:10.3390/nano9040497)
Supplement: Supplementary file 1 [file nanomaterials-09-00497-s001.pdf]

Supplementary material

Injectable Alginate-Peptide Composite Hydrogel as a Scaffold for Bone Tissue Regeneration

Moumita Ghosh †, Michal Halperin-Sternfeld †, Itzhak Grinberg and Lihi Adler-Abramovich *

Department of Oral Biology, The Goldschleger School of Dental Medicine, Sackler Faculty of Medicine, Tel Aviv University, Tel Aviv 6997801, Israel; moumita.ghosh1986@gmail.com (M.G.); michal4@mail.tau.ac.il (M.H.-S.); tzakhi@gmail.com (I.G.)

* Correspondence: lihiA@tauex.tau.ac.il; Tel.: +972-3-640-7252

† Those authors contributed equally to this work.

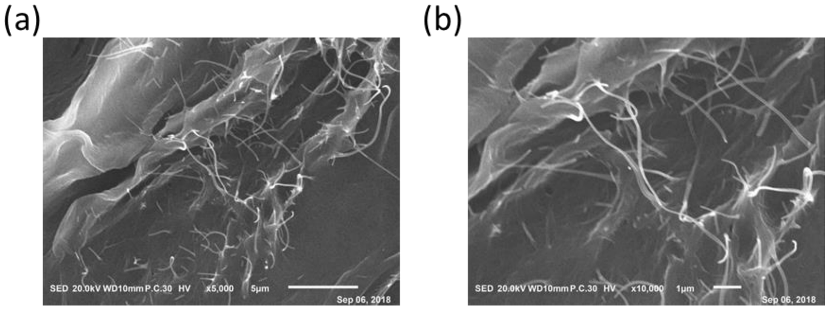

Figure S1. Scanning electron microscopy micrographs of Alginate/FmocFF composite hydrogel (a) scale bar = 5 μm, (b) scale bar = 1 μm.

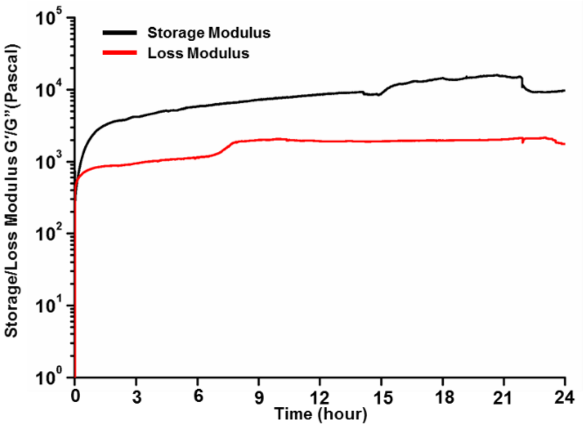

Figure S2. *In situ* time sweep oscillation measurements of Alginate/FmocFF composite hydrogel showing the storage modulus (G') and loss modulus (G'').

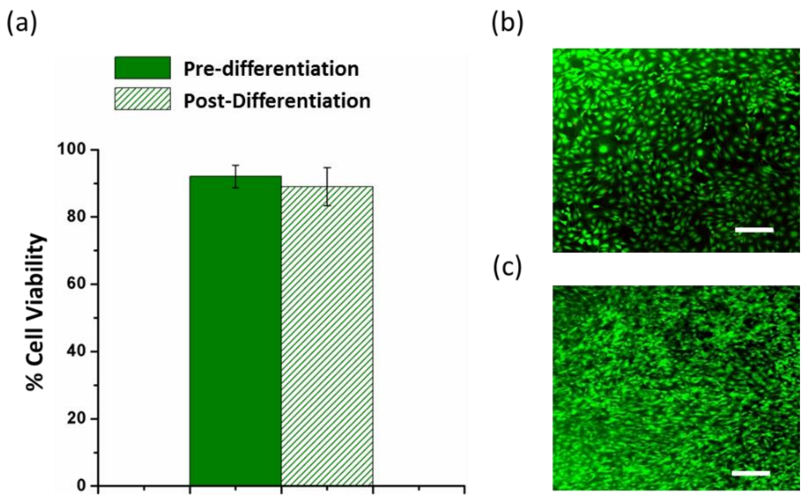

Figure S3. (a) Viability of MC3T3-E1 preosteoblast cells 3 days after seeding on the Alginate/FmocFF composite hydrogel and following 14 days of osteogenic differentiation. (b-c) Live/dead staining assays of MC3T3-E1 cells incubated for (b) 3 days on the composite hydrogel and (c) following 14 days of osteogenic differentiation. Scale bar = 500 m.

Gene name	Primer sequences (5′-3′)
Actin (House keeping gene)	F: 5′-CCGTCAGGCAGCTCATAGCTC-3′<br>R: 5′-GTCACCCACACTGTGCCCATC-3′
Alkaline phosphatase (ALP)	F: 5′- GGAATACGAACTGGATGAGAAGG-3′<br>R: 5′- GGTTCCAGACATAGTGGGAATG-3′
Osteocalcein (OC)	F: 5′-AGGACCATCTTTCTGCTCACT-3′<br>R: 5′-GCGTTTGTAGGCGGTCTTCA-3′
RUNX2	F: 5′-TGCACCTACCAGCCTCACCATAC-3′<br>R: 5′-GACAGCGACTTCATTCGACTTCC-3′
Collagen 1 (Col 1)	F: 5′-TGTTCGTGGTTCTCAGGGTAG-3′<br>R: 5′- TTGTCGTAGCAGGGTTCTTTC-3′
Bone morphogenic protein 2 (BMP2)	F: 5′-GCCGGTGTCCCTAATCTTT-3′<br>R: 5′- AGCCCTGTCTTATTCATCCA-3′

Table S1. Primers used for PCR amplification.
